# Hyperdiagnosis: Nosophobia and the Rise of Health Anxiety among Medical Students in the Age of Social Media and Artificial Intelligence

**DOI:** 10.31729/jnma.v63i290.9199

**Published:** 2025-09-01

**Authors:** Apeksha KC, Abhiyan Sharma

**Affiliations:** 1 Nepal Medical College Teaching Hospital, Atterkhel, Kathmandu, Nepal

**Keywords:** *anxiety*, *artificial intelligence*, *hypochondriasis*, *medicine*, *nosophobia*, *social media*

## Abstract

Widespread availability of health-related information via social media and artificial intelligence tools is contributing to an emerging pattern of hyperdiagnosis. Individuals, particularly medical students, develop heightened health anxiety and excessive fear of illness despite the absence of clinical disease. Nosophobia, which is a part of Medical Student Syndrome, is worsened by digital symptom-checking and constant exposure to disease content on social media.

## INTRODUCTION

Medical students often suffer from “medical student syndrome,” where they label any symptom they have as the disease they are studying.^1^ With the rise in the use of artificial intelligence (AI) tools and social media, increased exposure to information is even more likely to trigger irrational fears, worsen health anxiety, and create a pattern of attributing symptoms to oneself, often referred to as nosophobia or hypochondria.^1,2^ This results in medical students in “hyperdiagnosing” disease conditions in the absence of their required clinical or diagnostic criteria.

In this article, we explore how digital information, AI, and social media content contribute to nosophobia and cyberchondria, resulting in hyperdiagnosis among medical students.

## HISTORICAL RELEVANCE OF HEALTH ANXIETY

Health anxiety is not a newly emerging concern. It has been the driving force of medical advancements for centuries. During devastating outbreaks like the plague or smallpox, widespread anxiety pushed physicians to move beyond myths and superstition. Hence, they sought a real understanding about disease through observation and study. In the 1500s, King Charles IX of France was so paranoid about dying young that he constantly sought new treatments obsessively. It is said that many medical pioneers like Hippocrates were also motivated by their personal or societal health anxieties.

We don’t have to venture that far in history to understand health anxiety. During the COVID-19 pandemic, lack of information, misinformation and uncertainty fuelled health anxiety not just among common people but also among doctors. Anxiety disorders increased by 25.6% globally during the pandemic.^3^ Many resources of governments and organizations like WHO aimed at relieving heightened health anxiety among people, especially during lockdowns. Eventually, this also played a role in accelerating vaccine development. This illustrates that throughout history, fear of illness and the unknown has fueled medical advancements and ultimately laid the foundation for modern medicine.

## DIGITAL AGE, NOSOPHOBIA AND ITS COGNITIVE MECHANISMS

Hyperdiagnosis, in general terms, means overdiagnosis in the absence of physical symptoms adequate to meet the diagnostic criteria of a disease. “Nosophobia” is the fear of contracting a particular disease, while “hypochondria” refers to more general or persistent fears about illness.^4,5^

Today, the use of social media and search engines is so widespread that hundreds of health influencers create content about health and disease. Every symptom or disease is just a simple Google search away. On top of that, AI-driven algorithms are solely designed to capture our attention. Content about medical conditions is just unavoidable, and so is the health anxiety that follows. A student who just read a post about someone having leukemia might suddenly think their fatigue is a sign of cancer, even though it’s more likely due to stress or lack of sleep. That lump on their hand? Definitely a tumor. That palpable lymph node? Definitely tuberculosis. That headache that won’t go away? Definitely a chronic subdural hemorrhage. Medical students often tend to blatantly ignore how rare some serious diseases are (base-rate neglect) and instead focus on more memorable, recent examples (availability bias).^6,7^

The use of AI tools like ChatGPT and Gemini among medical students is very common these days. Medical students use them not just as an invaluable study resource, but also for looking up every little symptom they experience. A student watches five reels about ADHD and thinks, “Wait- I have all these symptoms,” then asks ChatGPT for confirmation. They end up believing they may need medication despite never being evaluated. Both AI and search engines confirm their fears (confirmation bias). This creates of a positive feedback loop of searching up symptoms, cherry-picking the evidence that they have the disease, ignoring contradictory or reassuring evidence, and receiving similar content through algorithms and cookies.^8^

This is worsened by the inherent nature of medical students, who are exposed to various cases during clinical postings, lectures, or just dreadful stories from professors. Medical students who have not studied sufficient medical resources or practiced medicine clinically have incomplete information about diseases and its real prevalence in the population. This leads to irrational fears of not just disease but of safe substances or behaviors. Seeing and learning dramatic cases in lectures or on social media feels like they’re more likely to happen to a student, and they’re convinced they’re suffering from it, even if it is statistically improbable.^7^ Almost every medical student experiences Medical Student Syndrome, a type of hypochondriasis causing health anxiety and fear in medical students that they have every disease they are studying.^9^

Students often believe that searching for symptoms will reduce their anxiety and provide them with a sense of control. But this can often backfire. Due to stawed beliefs such as “worrying helps me prepare” or “I’ll feel better if I keep searching,” they may fall into a pattern of compulsive and repetitive online searches, which ultimately worsens rather than relieves health-related anxiety.^10^

## PSYCHOLOGICAL AND ACADEMIC IMPACT

Though seemingly harmless, hyper-diagnosis may significantly affect medical students’ mental health, including increased anxiety and heightened stress.^10^ The constant fear of having multiple serious disease conditions is reinforced by compulsive searching on the internet, frequently asking AI tools or algorithms pushing similar content. This may lead to cyberchondria, a form of health anxiety caused by internet use.^11^ Binge internet searching for health information may lead students to believe that they can diagnose their issues based on search results, which may delay seeking professional help and increases the chances of self-medication.^4^ Persistent anxiety and increased stress affect concentration, memory, and decision-making, affecting students’ ability to focus on their studies properly eventually causing academic decline.^12^ Students suffer more when their online self-diagnosis and medical expert opinion do not align.

## RECOMMENDATIONS

Nosophobia and cyberchondria are both serious problems that cause health anxiety. But, this does not mean that we should attribute every genuine symptom that we have to them. Medicine as a career, especially as a student, is undoubtedly stressful, and getting sick is a very real possibility. If we experience symptoms that are recurrent or persistent, causing significant stress or morbidity, they should definitely be thoroughly investigated and treated accordingly under a doctor’s care.

As we discussed above, irrelevant fears exist, especially in medical students. Hence, medical schools should include sessions on nosophobia, and also make sure to teach students the actual prevalence of diseases, which helps us better navigate our fears, normalize these experiences, and reduce stress. We should always assess the credibility of any information online regarding health and diseases before blatantly believing it to avoid misinformation. Also, if medical colleges provide affordable and quality psychological services for students, it could help us open up and speak up about these problems and perhaps seek help for these symptoms. Applying stress management techniques like mindfulness and journaling or even attending cognitive behavioral therapy may also help manage these stressors.

## CONCLUSION

The growing dependence on digital information has transformed medical education, but also resulted in various psychological consequences, which lead to hyper-diagnosis of benign symptoms by medical students. This often leads to unnecessary stress, which affects their psychological state and academic performance. Hyper-awareness doesn’t always mean better awareness, and we must learn where to draw the line.
